# Comparison of the Purchasing Behaviour of Polish and United Kingdom Consumers in the Organic Food Market during the COVID-19 Pandemic

**DOI:** 10.3390/ijerph19031137

**Published:** 2022-01-20

**Authors:** Julia Wojciechowska-Solis, Aleksandra Kowalska, Milena Bieniek, Monika Ratajczyk, Louise Manning

**Affiliations:** 1 Faculty of Agrobioengineering, University of Life Sciences in Lublin, Akademicka 13, 20-950 Lublin, Poland; 2 Institute of Economics and Finance, Maria Curie-Skłodowska University, pl. Marii Curie-Skłodowskiej 5, 20-031 Lublin, Poland; aleksandra.kowalska@mail.umcs.pl; 3 Institute of Management and Quality Sciences, Maria Curie-Skłodowska University, pl. Marii Curie-Skłodowskiej 5, 20-031 Lublin, Poland; milena.bieniek@mail.umcs.pl (M.B.); monika.ratajczyk@mail.umcs.pl (M.R.); 4 School of Agriculture, Food and the Environment, Royal Agricultural University, Stroud Road, Cirencester GL7 6JS, UK; louise.manning@rau.ac.uk

**Keywords:** healthy food choices, sustainable food consumption, organic products, COVID-19, information sources, buying motives

## Abstract

The main objective of the study was to compare young consumer purchasing behaviour towards organic food in Poland (PL) and the United Kingdom (UK), countries with different levels of organic market maturity. The study was conducted by means of an online survey questionnaire during the COVID-19 pandemic between December 2020 and February 2021. The sample consisted of 862 PL and 161 UK consumers. 31% of PL respondents and 58.4% of UK respondents indicated they purchase organic products. Descriptive statistics, the Mann–Whitney U test and the two proportion Z test were used for statistical analyses. The results indicate that young consumers pay particular attention to the freshness and quality of consumed products. Concern for their own health and that of their loved ones, as well as the desire to eat better-quality products were the main motivations for the respondents to purchase organic products. Organic vegetables and fruits, eggs, dairy products, and meat and meat products, were among the most frequently purchased products in the studied cohorts. Experts (e.g., a dietitian, physician) were declared to be the first source of information concerning food products for young consumers. Next, family members were indicated. Social media content (PL respondents) and information from websites managed by institutions (UK respondents) were mentioned as the third source. UK consumers preferred short supply chains. The present study can be used by government bodies and companies to select the most effective communication channels for education and advertising and to develop effective commercial strategies aimed at young consumers.

## 1. Introduction

The pursuit of sustainable development is founded upon the adoption of the principles of agro-ecology and the reorientation of production systems, where activities contribute to the effective protection of the natural environment through the use of low-emission production processes [1]. Environmentally Sustainable Food Consumption (ESFC) is the use of food products “that respond to basic needs and bring a better quality of life, while minimising the use of natural resources, toxic materials and emissions of waste and pollutants over the life cycle, so as not to jeopardise the needs of future generations” [2,3]. Sustainable consumption connects with the growth of responsible and ethical consumption [4]. Ethical consumption is the practice of buying items not solely for economic reasons, but instead based on moral and personal beliefs and social factors [5,6]. Thus, through their choices, consumers can shape the demand for food from a specific place of origin, produced in a specific production process, or supplied by producers who voluntarily take into account sustainable development standards, i.e., geographical indications, local brands or organic farming standards [7].

In recent years, people have become more concerned about the degradation of the natural environment [8,9] and the state of their own health [10]. This concern has led to a change in behaviour of individuals who seek alternatives to the consumption of conventional food products. Even before the pandemic, there was a noticeable surge in concern for the environment and sustainable development. The COVID-19 pandemic has further accelerated this process as consumers have begun to purchase food more consciously, choosing products that positively affect health [11]. Therefore, the health concern caused by the ongoing pandemic may contribute to an increase in the purchase and consumption of organic food, i.e., goods that are produced using organic farming methods and products processed without the use of chemical additives and preservatives [10].

Organic production plays a fundamental role in achieving the Sustainable Development Goals (SDGs) of the 2030 Agenda. It is estimated that organic farming is pursued by around 3.1 million producers in approximately 190 countries covering 73.4 million hectares (ha). In 2019, only 1.5% of total agricultural land in the world was organic [12] but organic farming is one of the fastest growing sectors of global agriculture [13] which is evidenced by the fact that the share of organic farming area in the total global Utilized Agricultural Area (UAA) has doubled over the period 2010–2019 [12]. The total share of the organically managed area was estimated at 8.5% of UAA in the European Union (EU) in 2019 [12]. The EU Common Agricultural Policy (CAP), which has offered financial support through agri-environmental and rural development programmes for the past thirty years or so, has significantly contributed to expanding the organically managed farms and areas under organic production in the EU countries [14,15]. However, there are considerable differences regarding the proportion of agricultural land under organic management across the EU, between the new EU member states and the original EU-15. Poland (3.5%), the United Kingdom (UK) (2.6%) and many other European countries were far below the 2019, EU average [12]. The UK was still counted as an EU member state in 2019 statistics of the Research Institute of Organic Agriculture FiBL. Furthermore, the organically farmed area decreased by 2.7% in Poland and by 34.4% in the UK over the period 2010–2019 [12]. Although organic farms in the EU-15 were financially supported under the EU CAP for much longer time than the farms in new EU member states, the pace of organic agriculture development has differed across all the EU countries. Hence, the issue of fostering the development of organic farming is complex. Some studies suggest that the factors impeding the growth of organic farming have included: management-related factors, national policy on organic agriculture, cultural barriers, and market uncertainty [16]. It is clear that dynamic and sustainable development of organic farming requires an effectively functioning market of organic products. In 2019, the value of this market was estimated at approximately EUR 106.4 billion globally, with the largest share taken up by the United States (US) and EU member states [12]. In countries such as the US, France and Germany, that have the largest proportion of consumption of organic food products, the market is characterised by high internal demand, effective distribution channels and a well-developed sphere of organic food processing, which drives profitable sales for organic farms [17,18,19]. In 2019, the UK was the eighth country worldwide in terms of organic food market, being worth in the UK about EUR 2.679 billion. Although, it constituted only 1.8% of the UK food retail sales. At this time, the Polish market for organic food was relatively small (EUR 0.314 billion) and constituted only 0.6% of total food retail sales. Since the COVID-19 pandemic creates both challenges and opportunities for the food system, increasing uncertainty and rapidly changing conditions during the pandemic are becoming the standard environment for humanity, and food-related consumer behaviours are now worth exploring.

Understanding the eco-friendly consumer behaviour of Generations Y and Z on the food market is crucial in designing a green marketing strategy [20,21,22]. There are 1.8 billion Generation Y consumers (Millennials) [23] and almost 2 billion Generation Z consumers globally [24], and these numbers are still growing. We perceive anyone born from 1981 onward as a young consumer [25], with those belonging to Generation Y (born between 1981 and 1996) or Generation Z (born from 1997 onward) having much in common [26]. Representatives of Generations Y and Z are technologically proficient and digitally hyper-connected, hence the online environment (in particular social media) is a good space for marketers to communicate with numerous consumers, regardless of their geographical location [27]. Moreover, these generations are more diversified in terms of racial and ethnic origin than others [28]. Finally, as a result of Internet access, this consumer group has lower brand loyalty, creates new habits more easily and is more willing to change their styles and means of communication [11,29]. According to researchers, consumers of Generations Y and Z are usually enthusiastic about purchasing organic products [30,31]. The majority of studies pertain to consumer behaviour in the markets of developed countries such as Germany [32], Australia [33], or Japan [34]. Studies concerning consumer behaviour in emerging markets such as Vietnam [35], China or Brazil [36], Tanzania [37], Turkey, Pakistan and Iran [38] are also readily available. However, there are few studies that compare countries with a mature organic food market with those where the organic market is still under development [15,39,40]. The PL organic food market is considered here as still at the developing stage, as the use of organic food products began much later than other European countries such as the UK, where the market for organic products is more mature [39].

The objective of the study was to compare consumer purchasing behaviour concerning organic food in two countries—the UK, where the market for organic products is mature, and PL, where the market is developing. The research sample consisted of young consumers of Generations Y and Z from the aforementioned countries and the data was collected during the COVID-19 pandemic. The general research question considered in this study was:

Are there differences between young consumer behaviour concerning organic food in PL and the UK, as related to health consciousness?

The authors aim to answer the following research questions:

RQ1: What does healthy eating denote for young people during the COVID-19 pandemic?

RQ2: Where does the young consumer obtain information concerning food products they purchase?

RQ3: What information regarding food products is important to the young consumer?

RQ4: Do young consumers from PL and the UK who prefer to purchase organic food constitute a homogeneous group in terms of their attitude to organic food?

RQ5: What motivated young consumers to purchase organic products?

RQ6: Which products labelled as organic food are the most frequently purchased by young consumers?

The study exploited secondary data from the literature, available statistical data and empirical material collected using a structured questionnaire (see Supplementary Materials). The article is structured as follows: first, in Section 1, there is an introduction to organic food consumption research. Section 2 includes a literature review of consumer demand for organically produced foods on the global market. Section 3 considers the methodological approach. Section 4 provides the results of analysis of the empirical study conducted among young consumers in PL and the UK. Section 5 draws the discussion together and Section 6 concludes the paper and seeks to identify differences in the perception and purchasing behaviour of young consumers on the organic food market in PL and the UK.

## 2. Literature Review: Consumer Demand for Organically Produced Foods

Organic food is perceived to benefit consumers and the environment. It also supports the local economy [41,42]. Consideration of social, economic, and environmental issues linked to creating sustainable development benchmarks translates into a more credible image of food producers in the eyes of their customers. In fact, innovation in organic products has been recognised as a strategic opportunity for several aspects, including increased profits, environmental sustainability, and a better quality of life for local producers and consumers of organic products [43]. Studies referred to by Verain et al. [44] distinguish such groups of consumers as: the “green segment” characterised by self-improvement and openness to changes, with an observable tendency for purchasing ecological products and caring for the environment; “potentially green consumers” who care about the environment and purchase organic products, but who are at the same time sensitive to price increase, and “non-organic consumers” who are driven more by achievements and tradition than ecological motives.

Organic production exploits environmentally friendly farming practices and supports a high degree of biodiversity and the protection of natural resources. In addition, it maintains high standards of animal husbandry and uses production methods that meet the requirements of consumers who prefer products made with the use of natural substances and processes. Therefore, this ecological method of production performs a double social function. First, it is a system that exerts a positive impact on the natural environment, which contributes to the emergence of broadly understood agri-environmental benefits. Secondly, organic farming responds to the changing structure of market demand. When consumers select organic products, they usually pay a higher price for them than for products that were not produced using such methods [45]. As stated by Łuczka and Kalinowski [46], the organic food to conventional food price-ratio in the EU can be strongly diversified, reaching in some countries to over 300% for certain products. This depends, inter alia, upon the maturity of the market, the supply-demand relationship and distribution channels. As a result of PL’s accession to the EU, subsidies promoting organic farming were introduced. This translated into a dynamic increase in the acreage, an increase in the number of organic farms, and an increase in production. This enabled the needs of the internal market to be satisfied and the export of organic agri-food products to countries where the internal demand significantly exceeds national supply [15].

In Europe, the greatest level of organic product consumption is in countries such as Germany, France, and Italy, where there is a relatively long tradition of organic farming and a high level of economic development, which translates into a higher level of income in some sectors of the community, thus making organic food affordable. The largest organic food markets in the world include the US, Germany, France, and China [12]. The average European per capita expenditure on organic food in 2019 was EUR 55.8. The value of the market per capita varies by country. The highest per person organic food purchases were noted in Denmark, Switzerland, Luxembourg, Austria, and Sweden, with average spending of EUR 214–344 per person per year. Consumer spending on organic food in the UK has increased by 25% in the last decade. However, in 2019, it remained below the European average at EUR 40 per capita per year [12]. Eastern European countries show the lowest per capita expenditure on organic food. The average PL consumer spent EUR 8 per annum on organic food in 2019. This shows that although the UK has a well-established organic food market, consumers from the UK and PL are reticent purchasers of organic food among Europeans [25].

The time of the pandemic exerted a positive impact on the development of the aforementioned market. Consumer behaviour in the organic products market has changed during lockdown. In the UK, a rise in demand during ‘lock-down’ drove sales of organic produce in the UK to rise by 12.6% to £2.79 bn in 2020 [47]. UK consumers seem to have become more health- and provenance-conscious over the pandemic leading to a rapid increase in the sales of organic food and drinks [48]. Thus, there is a need to continue promoting informed consumer choices on the organic food market to increase demand for organically produced food in countries where there is a relatively lower interest in such foodstuffs.

Researchers often emphasize the positive impact of appropriate labelling of organic products upon the consumer’s purchasing intentions, e.g., the organic production logo, designation of the certifying body, location of the production of unprocessed agricultural products [49,50]. Thus, confidence in organic food is built by positive experiences related to the high-quality assessment of such food, confirmed by an independently verifiable certificate. The confidence in the organic food production process, including standards and controls, is a strong determinant of purchasing decisions [51,52]. According to reports by Paul and Rana [8], a clear and visible label on a product is a prerequisite for organic food products being selected.

Consumer behaviours that focus on health and environmentally friendly attitudes can contribute to sustainable economic development, social progress and an improved quality of life. Sustainable economic development includes, in addition to economic growth, a series of quantitative, structural, and qualitative transformations that meet current food needs without compromising the needs of future generations [53]. The level of consumer health awareness determines their attitude to potential health problems, i.e., the willingness to take action in favour of health protection [54,55]. There is a widespread belief that organic food is healthier because it is nutrient-rich and chemicals-free [56], and consumers are willing to pay for this [57,58]. Health awareness is, therefore, a critical factor in selecting organic food [59]. Empirical data show that health awareness has a beneficial effect upon purchasing and consumer’s purchasing intentions. Consumers who are concerned about their health are more likely to purchase organic food than food cultivated by conventional or other non-organic methods [8,60,61,62].

## 3. Materials and Methods

The study was exploratory in character as its purpose was to compare consumer purchasing behaviour in the organic food market in countries with different maturity of the organic food market. The empirical study was conducted in PL and the UK during part of the global coronavirus pandemic, i.e., in the period between December 2020 and February 2021. Very severe national restrictions were in place in this period. The restrictions related to movement and gathering and were connected with the spread of the COVID-19 pandemic in a “hard lockdown”. The level of lockdown and the types of restrictions introduced at the time were different in the UK and PL, which was a limitation of this study. The main research centres where the study was conducted were the Maria Curie-Skłodowska University (UMCS) in Lublin (PL) and the Royal Agricultural University (RAU) in Cirencester (UK). The survey questionnaire was approved by the Research Ethics Committee at both universities. The Polish and UK sample sizes were different which partly resulted from the fact that the number of students at UMCS (ca. 20,000) is significantly different from the number of students at RAU (ca. 1100). However, direct comparisons were considered valid see [63]. The nonparametric tests were used where equal groups are not mandatory. Moreover, the two-proportions Z test corrects the differences in the sample sizes [64,65].

The structured questionnaire had two language versions—Polish and English, each dedicated to a specific country (PL/UK). An online survey questionnaire (CAWI) was used in the study. The survey was conducted by means of the 1 KA online survey tool. The recruitment criteria for the study were the consumer’s legal age and being a representative of either Generation Z (18–23 years of age in 2020) or Generation Y (24–39 years of age in 2020). The age ranges in each cohort were the same as in previous research/empirical studies [25,66,67]. The questionnaire was distributed among students by means of the universities’ internal e-mailing systems after obtaining consent from the authorities of the above-mentioned universities in PL and the UK. The convenience sampling approach was not designed as being probability based and the use of the convenience sampling approach is considered when interpreting the findings of the study. In a few cases, the questionnaires had certain deficiencies as participation in the study was completely voluntary and the provision of answers to the questions was technically unforced (the option of skipping to the next question without answering a question was open). The analysis was undertaken only on completed questionnaires (see Supplementary Materials).

In order to answer the research questions from the data derived, the calculations were carried out using the SPSS software (ver. 26). Descriptive statistics, the Mann–Whitney U test and the two proportion Z test were used in the analyses. The Mann–Whitney U test is dedicated to compare differences between two independent groups if the dependent variable is not normally distributed and it is either continuous or ordinal. Usually, under specific assumptions, the test examines if there is a significant difference between the medians of those two groups. Two-proportion Z test is used to determine if the difference between two proportions between groups is significant based on the assumption that the groups are independent of each other [64,65].

## 4. Results

There were 973 completed responses to the survey, which form the dataset that is considered in this section of the paper. In PL, the questionnaire was completed by 812 respondents, and in the UK by 161 (Table 1). In total, 1667 respondents from both countries started the survey. The completion rate was 58.3% and this in part may be due to the extensive nature of the survey. The average time the respondents devoted to completing the questionnaire was 16 min 38 s. Nearly three quarters of respondents altogether in both groups in PL and the UK were female, two thirds of respondents in PL were from Generation Z (66.1%) and the converse in the UK 62.1% were from Generation Y. Eighty-four percent of the respondents in the PL group (84.2%) were students and this fell to 62.1% in the UK group. When asked their perceived financial status only 7.1% of PL respondents and 9.3% of UK respondents stated that it was ‘bad’ or ‘very bad’. More respondents in the UK group (19.9%) stated they had a food allergy than in the PL group (16.6%). There was proportionally more national diversity in the UK group than the PL group where 93.7% of the PL respondents defined themselves as PL with less UK respondents (65.2%) describing themselves as UK citizens.

The study aimed to answer the six above research questions, which will be considered in turn in the results section.

**RQ1: What does healthy eating denote for young people during the COVID-19 pandemic?**

The time of the pandemic can be labelled as a period of redefinition of consumer behaviour, especially with food markets and it can be expected that changes in purchasing preferences have occurred in every economic sector and continue to do so. It should be emphasized that, data suggests that perhaps due to the re-evaluation of health aspects during the pandemic, the market for organic products has grown, especially on developing markets such as PL [10,68].

At the beginning of our study, respondents were requested to define what the term “healthy eating” meant to them. The three most frequently indicated interpretations of this concept in both groups from PL and the UK were the same, i.e., healthy eating for young consumers primarily denotes the following: “ensuring the freshness of my food” (a more important feature for PL respondents than those in the UK), “making sure I eat foods that are good for me and avoid foods that are bad for me” and “ensuring the quality of my food” (Table 2).

The Mann–Whitney U test showed that there are statistically significant differences in six interpretations of the term “healthy eating” between the groups (Table 2). For example, food safety was more important as a factor for UK respondents than for respondents from PL. Ensuring freshness of food is paramount for both groups (as indicated by the high averages). However, it is more important for PL respondents (higher average ranks in the Mann–Whitney U test). For the UK respondents, the following were considerably more important in the context of healthy eating: “ensuring the appropriate calorific balance of my food intake”, “ensuring the appropriate nutrient balance of my food intake”, “trying to reduce the environmental impact of my food choices” and “ensuring that my diet is adapted to lifestyle” (higher average ranks in the Mann–Whitney U test and statistically significant differences at *p* < 0.001). Provenance of food was ranked as of interest, but there was no statistically significant difference in response between PL and UK groups.

These results confirm the greater maturity and food awareness of young UK consumers in the sample population, which is expressed through a more multifaceted perception of the term “healthy eating”—not only in relation to personal health, but also in the context of environmental protection.

**RQ2: Where does the young consumer obtain information concerning food products they purchase?/RQ3: What information regarding food products is important to the young consumer?**

Consumer engagement with information is inextricably linked to the flow of information between them and other entities in the surrounding environment. The study requested respondents to identify sources of information related to the foods they consume (scale adapted from [69]). For this purpose, respondents were asked to indicate three sources, ranking them as first, second and third choice (rank scale).

Experts, e.g., dietitians, physicians, were the chief source of information concerning food, diet and nutrition for respondents from both groups. Forty-three percent of respondents from the UK and over a quarter of the respondents from PL indicated experts as the main source of information. Family members were ranked second in importance by all respondents. Social media (PL consumers) and information from websites managed by institutions (UK consumers) ranked third. The significance of information from family, friends, mass media, professional literature, and newspapers and magazines is similar in both groups. The Z test did not indicate differences between the groups of PL and UK respondents in this respect (Table 3). However, it was evident that in the mature market (UK), far more young consumers placed higher trust in experts (a source indicated more frequently both in first place and in one of the first three places in terms of its importance for respondents). On the other hand, in the developing market (PL), more young people access social media content (source indicated more frequently both in the first place, as well as in one of the first three places in terms of its importance for the respondents).

Additionally, the respondents were asked questions concerning the importance of certain product attributes: the provenance of the product (national, regional), organic certification, food produced in a traditional manner and the distance the purchased product travels from the manufacturer to the location of sale. PL and UK respondents again differ in their response on this issue (Table 4). Almost all of these characteristics are far more salient for UK respondents (higher averages) compared to those from PL. The most important product attributes for PL respondents were that the food is produced in PL, more so than the specific region, the food is produced in a traditional manner and that the food is organically certified (highest averages). For the UK respondents, the most significant characteristic is the provenance of the product (made in the UK), the distance the food travels from the producer to the location of sale, and whether the food originates from the respondent’s region of residence and then whether the product is produced in a traditional manner or is organically certified. UK respondents are probably aware that due to the insular nature of the country (it is an island), many food products are imported. As a consequence, it is likely that the distance the product travels to arrive on the table is more important for them than for PL respondents (*p* = 0.000; average ranks higher in the group of UK respondents). Another reason may be the higher level of environmental awareness reported by the UK respondents and this may heighten their concern over food miles.

The PL market for organic products is a developing market, while the UK market can be considered mature. The UK respondents were as a result more familiar with the advantages of organic products than PL respondents. However, PL producers have recently been dynamically entering the market for organic products, which a few years ago was considered a niche [70]. One of the factors determining the increase in the interest in organic products is economic growth, and thus the increase in societal income. Numerous educational, information and media campaigns in PL are supported by government programmes that help to disseminate knowledge about the health-promoting characteristics of organic products [71], however the per capita spend on organic food remains low.

**RQ4: Do young consumers from PL and the UK who prefer to purchase organic food constitute a homogeneous group in terms of their attitude to organic food?**

**Figure 1:**
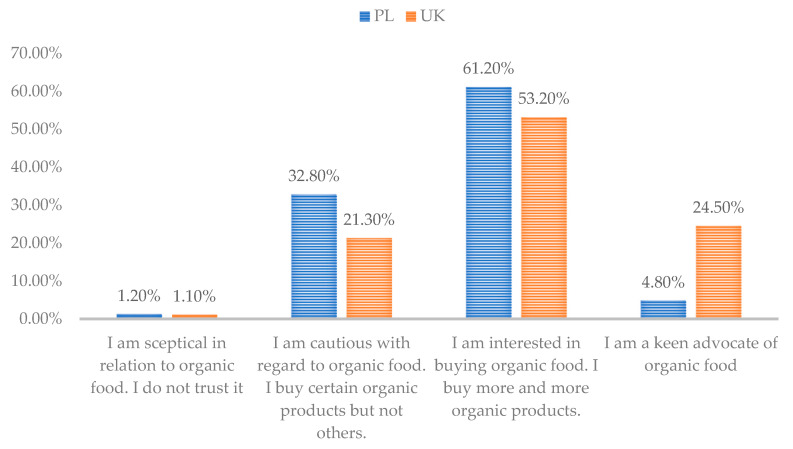
Attitude towards organic products among people purchasing organic food in the surveyed countries.

Consumers from both surveyed markets differ in the organic food purchasing behaviour. In the PL group of respondents, 31% of young consumers declared they purchase organic food, while in the UK this rises to 58.4% of respondents. Among respondents from PL and the UK, who declared they purchased organic products, a different attitude towards this type of food can be observed (Figure 1). Of those who report they purchase organic food the degree of preference varies between PL and UK respondents. Young UK respondents have a stronger preference for purchasing organic food than consumers of the same age in PL. Almost one quarter of the surveyed UK respondents (24.5%) who purchase organic food describe themselves as “keen advocates of this type of food” compared with just under one in twenty (4.8%) of PL respondents (Figure 1). In both groups, the dominant positive attitude is an “interest in, or an increasing interest in, purchasing organic food” (61.2% in the PL group and 53.2% in the UK group). Young respondents in the surveyed countries include people who increasingly purchase organic food. In terms of those who report themselves as being “cautious” (32.8% in PL group, 21.3% in the UK group), they state they “buy certain organic food products and not others”. Only just over 1% of both groups described themselves as “sceptical” and not trusting of organic food. This data suggests that the young consumers across the sample are not a homogeneous group with regards to their purchasing organic food. Both in PL and in the UK, the representatives of Generation Z and Y differ in the attitude towards, knowledge or beliefs in relation to organic food. There is more interest and a stronger interest in the UK group compared to the respondents from PL. This means that despite the declared purchases of organic food, the structure of their shopping basket (the number of products purchased as organic) differs. At the same time, these data confirm that the UK market is more mature in the context of organic food purchases.

Researchers frequently seek evidence for the motivations that prompt consumers to purchase certain foods. The acquired information helps to frame the factors that influence demand in the market and to assess the development prospects of this market. Identifying the factors motivating the consumer to purchase organic products helps producers to effectively pursue promotional activities and assists governments in running social campaigns if they are seeking to change consumer behaviour.

**RQ5: What motivated young consumers to purchase organic products?**

The survey items regarding the motivations for purchasing organic food were adapted from other studies [72,73]. Advertising campaigns for enterprises and social campaigns can increase market size by encouraging the consumer to purchase a specific product. Campaigns frequently perform an educational role raising the consumer’s knowledge of the nutritional and environmental values of food products (Table 5). It should be emphasized that respondents in both groups most frequently mentioned the following motivations for choosing organic food: “concern for their own health and/or that of their loved ones”, “desire to eat higher quality products” and “care for the natural environment”. However, comparing the averages between groups (PL vs. UK respondents), it is evident that for young UK consumers, environmental issues (“taking care of environment”) are more important in making decisions about purchasing food products than for young PL consumers (statistically significant difference—see Table 5). Indeed, environmental issues were the most important factor for UK respondents. On the other hand, such issues as “concern for one’s own health and/or that of loved ones” or “the desire to eat higher quality products” do not diversify the studied groups.

However, PL and UK respondents are diversified (Table 5) by such motivations as “the desire to try something new”, “the desire to lead a specific lifestyle” and “the impact of social media content”, which in each case offered a stronger motivation to purchase organic food in the PL respondents (higher average ranks in the Mann–Whitney U test). This shows that in developing markets, issues such as the trend for leading a certain lifestyle and/or particular nutrition, including imitation of others (inter alia under the influence of influencers especially social media influencers), as well as having a greater openness to novelties among young consumers, means there is a noticeable increase in the attitude of a ‘trysumer’, i.e., a consumer who is keen on testing novelties [74] and these factors may be more important than the level of environmental awareness. As a consequence, this means that in developing markets, consumers may need to be educated far more about the social and environmental values of organic farming.

**RQ6: Which products labelled as organic food are the most frequently purchased by young consumers?**

Organic food producers recognise the benefits of the support for organic production from government agencies and EU institutions which comprise income support and publicly funded campaigns for the advertising of organic foodstuffs [71]. As a result, consumers can access a wide range of proposed organic products in both surveyed countries. However, it should be mentioned that the supply of organic products is largely complemented by food imports [73].

The present study reveals which certificated organic food products are purchased by respondents who declare they purchase organic food and which products are not selected by them in this product segment. This may mean instead choosing and purchasing conventional products or other “eco-friendly alternatives” in certain product categories, such as local or vegan food.

The respondents were questioned on how frequently they purchased each of the 19 organic product categories identified in the survey (Table 6). The analyses show that the respondents from both countries differ significantly in the frequency of purchases of organic products for nine food categories. For all categories, except groats that are not widely eaten in the UK, UK respondents purchase organic food more frequently. This shows that respondents in mature markets not only choose organic products more frequently, but also have a diversified organic food shopping basket. Regardless of the phase of market maturity, the following can be observed: products which are the least frequently purchased as organic products represent foods considered as unhealthy, or vice, categories—“wants” [75], i.e., beer, soft drinks, sweets and candy (lowest averages). Those foods reported as being purchased most frequently (for both groups) encompass fresh fruits and vegetables and eggs (highest average). This finding supports the work of Denver and Christensen [76], p. 9, states that “organic consumers are more likely to eat in line with Dietary Recommendations.”

## 5. Discussion

The main objective of the study was to compare the reported buying motives and behaviour concerning organic food in PL and UK, two countries where markets are at different maturity stages. The respondents were from Generations Y and Z. The research was undertaken during the coronavirus pandemic. The study examined the following: the respondent’s understanding of the term “healthy eating”, sources of information and the significance of individual information sources concerning organic food products, what motivates young consumers to purchase organic products as well as which organic products young consumers report they purchase most frequently.

Society tends to perceive organic food consumers as more moral, caring, generous, socially responsible and of a higher status than conventional food consumers [77]. Individual consumer choices impact the environment, sustainable development and health [78]. There has been a recent shift in global consumer trends towards a more balanced and healthier diet through the consumption of organic food [48,79], especially during the COVID-19 pandemic, and organic products are considered healthier and their production inherently less harmful to the environment [80]. This is confirmed by respondent responses in this research concerning what they understand by healthy eating. The health value of organic products has been emphasized on numerous occasions in research on various markets [17,53,73]. With the PL respondents, it seems that the nationwide educational campaign “Wiem co jem” (“I know what I eat”) brings tangible results [81]. Indeed, it is asserted that consumers increasingly pay attention to the products they buy, read the content of labels, and try to live in such a way as to minimise the negative impact of their purchasing decisions on the environment [82].

The studies of Aertsens et al. [83] show that the level of consumer knowledge influences the perception of a given product and is related to searching for detailed information about it. In particular, low levels of consumer knowledge, lead to a lack of confidence in the quality of products, motivating consumers to seek additional information [83]. As organic food is relatively new to markets such as in PL, compared to those in Western Europe, consumers may have limited knowledge of such food [84,85]. Similar observations were made about the organic products market in China [86].

As established empirically in this study, respondents primarily view “healthy eating” as the consumption of fresh, good-quality products that benefit them, and avoidance of those that could deteriorate their health (RQ1). This means that the consumers of Generations Y and Z were guided by health benefits when purchasing organic food. This finding concurs with previous studies [38,87,88]. Research conducted in Denmark indicates that social media has a significant impact on decisions about healthy eating among young consumers [89]. Findings in this work (RQ2) reach a similar conclusion. Social media content is an important source of information for young consumers in a developing market (PL). Additionally, studies conducted in Tuscany, Italy, a region rich in healthy eating traditions, show that the young generation take into account the opinions of family members, friends and news from social media, the most frequently [90]. However, there were differences between the responses, as UK respondents stated they were less influenced by social media in the study.

The influence of food provenance is currently a popular line of research. For an ethnocentric consumer, importing products from other countries is not considered appropriate, as it is not patriotic and, what is more, it would be harmful to the domestic economy and employment. PL and the UK are among European countries that strongly support the policy of domestic production, preferring short supply chains [91,92,93]. This research also confirms that for young respondents, both from PL and the UK, information about the country of origin of the product is one of the most important elements when it comes to organic food products (RQ3). However, this might be for different reasons as UK respondents were more concerned about the environment and food miles. This may not be for patriotic reasons, more for its environmental impact. Thus, shorter chains may be preferred for this reason and not because of provenance per se.

Young consumers who purchase organic food in PL and the UK are not a homogeneous group (RQ4). UK respondents operate in a mature market, with almost a quarter of respondents describing themselves as advocates of organic food. In PL, the developing market, when it comes to organic food products, nearly one-third of consumers who purchase organic food describe themselves as “cautious”. Numerous studies on organic food discuss its functional aspects, i.e., emphasize its importance for consumer health [57]. This research also shows that in developing markets, hedonistic values may be of significance (e.g., as a result of buying organic products, the possibility of creating a specific lifestyle, imitating others). However, the willingness to test new products may play an important role. This is a viable feature especially for the so-called trysumers (the consumer who tries to personally verify the market offer and is very open to new products) (RQ5).

According to van de Grint et al. [94], organic consumption is a relatively new phenomenon in the food industry. However, consumers have already developed their own purchasing preferences. When viewing markets in the same phase as the market in PL, i.e., Serbia, Romania and Slovakia, consumers most frequently voice a preference for the purchase of organic fruits and vegetables. The shopping basket of organic consumers of these countries also includes organic oils, nuts, eggs, milk, and dairy products [95,96,97]. Studies conducted by Hermaniuk [98], similarly to the research presented in this paper, emphasize that snacks, stimulants (beer) and various additives are not very popular among organic consumers (RQ6). The contents of the organic product basket indicate specific consumer preferences in relation to fruits and vegetables (the most-bought item in the surveyed countries) and may result from the availability of certain products on the market [17,42] and the propensity of organic food consumers to wish to promote a healthy lifestyle [76,99].

The market for organic products, both in Europe and worldwide, is likely to continue to grow rapidly [100]. Research on consumer behaviour on both mature and developing markets can help to create an appropriate information message emphasizing the rationale for purchasing organic food and its advantages.

Future empirical research may address the shift to online organic food shopping during the pandemic which was not addressed in this study. A thorough analysis of factors causing the differences in young consumer purchasing behaviour towards organic food in PL and the UK with the use of econometric models will be the subject of further research.

## 6. Conclusions

This study shows differences in the perception and purchasing behaviour of young consumers in the organic food market, and particularly, on the market characterised by a different degree of maturity in relation to organic food, i.e., the UK mature market and the PL developing market. In the mature market, in this study nearly two-third of respondents declare they purchased organic food, while in the PL market this reduces to around one-third (31%). UK respondents have a more positive attitude to buying organic food. In eight out of 19 product categories they purchase a given product more frequently as organic food than their PL counterparts. Additionally, understanding interpretation of the term “healthy eating” as well as motivations to purchase organic food may be influenced by more effective messages aimed at young consumers. Over the pandemic, consumers have become more conscious about health and environmental issues, which has stimulated interest in the organic food products market. The results presented may be useful in defining effective initiatives for the popularisation of health-promoting behaviours among young consumers of Generations Y and Z, e.g., in educational campaigns as well as promotional and motivational initiatives. Health and environmental concerns were reported as being considered more by UK respondents and their preferred sources of information were more likely to be specialists and experts. Thus, familiarity with informed, evidence-based sources of food and nutrition-related information for Generations Y and Z enables better decisions making. Taking further steps to develop the sustainable production, distribution, and consumption of organic food, as part of the wider EU Farm to Fork Strategy must include the provision of information and knowledge to the public concerning the benefits of eating organic food, in particular domestic/local food. This should also reduce environmental burdens arising from the food system and improve public health.

## 7. Limitations

The present study has certain limitations related to the time period in which it was conducted during the COVID-19 pandemic, which may affect the potential for wider generalisation of the findings. The time period had to be used because of the confines of the research grant schedule. Furthermore, the PL group in the sample was much larger than the UK group. The study was also limited to the cohorts of the Generation Y and Z, reducing opportunities for wider generalisation. The approach to conducting research using the CAWI method may seem to be a significant limitation in reaching a more diverse sample of respondents. However, the pursuit of this method was dictated primarily by the low cost of application.

## Figures and Tables

**Table 1:** Demographic structure of the sample from the UK and the PL populations.

Variables		PL	UK
Study sample	N	812	161
Gender	Female	72.5%	75%
	Male	27.5%	25%
Age	Generation Z	66.1%	37.9%
	Generation Y	33.9%	62.1%
Place of residence	Lublin/Cirencester	39.8%	19.9%
	Another town in PL/UK	58.7%	68.3%
	Another town outside PL/UK	1.5%	11.8%
Student status	Yes	84.2%	62.1%
	No	15.8%	37.9%
University	Maria Curie-Skłodowska University in Lublin/Royal Agricultural University in Cirencester	66.2%	45.9%
	Other	33.8%	54.1%
Perceived financial situation	Very bad (I am not able to meet my basic needs)	0.1%	1.2%
	Bad (I am only able to meet my basic needs)	7.0%	8.1%
	Average (I can afford to buy most of the things I would like to have)	53.5%	45.3%
	Good (I can afford to buy what I want)	28.1%	19.9%
	Very good (I can afford to buy what I want and save/invest some money)	11.2%	25.5%
Nationality	PL/UK	93.7%	65.2%
	Other	6.3%	34.8%
Food allergy	Yes	16.6%	19.9%
	No	84%	80.1%

**Table 2:** The meaning of the term “healthy eating” in the surveyed groups—averages and *p*-values in the Mann–Whitney U test for the differences between respondents from PL and the UK.

Response	Mean		*p*
	PL	UK	
Ensuring the freshness of my food	4.53	4.4	0.003 ***
Making sure that I eat foods that are good for me and avoid foods that are bad for me	4.32	4.37	0.095
Ensuring the quality of my food	4.3	4.26	0.414
Ensuring the safety of my food	4.06	4.21	0.012 *
Ensuring that my diet is adapted to my lifestyle (physical activity, occupation, etc.)	3.9	4.19	0.000 ***
Ensuring the appropriate nutrient balance of my food intake	3.84	4.28	0.000 ***
Ensuring the provenance of my food	3.73	3.62	0.143
Ensuring the appropriate calorific balance of my food intake	3.67	3.99	0.000 ***
Eating at appropriate meal times	3.57	3.7	0.085
Trying to reduce the environmental impact of my food choices	3.41	3.81	0.000 ***

Note: The average calculated on a rating scale from 1 (strongly disagree) to 5 (strongly agree). Statistically significant differences were highlighted in bold (* *p* < 0.05, *** *p* < 0.001). PL group: n = 812, UK group: n = 161.

**Table 3:** Prominent sources of food and nutrition-related information for the UK and PL samples—fractions of groups, two-proportions Z-test statistics and *p*-values.

Type of Information Source	N		Percentage		Z	*p*
	PL	UK	PL	UK		
**The most prominent source of information**						
Experts, e.g., dietitians, physicians	206	70	25.5%	43.5%	−4.6266	<0.00001 ***
Family	158	25	19.5%	15.5%	1.1854	0.234
Websites managed by institutions	131	16	16.2%	9.9%	2.0213	0.043 *
Social media, e.g., Facebook	106	3	13.1%	1.9%	4.1238	<0.00001 ***
Bloggers	53	3	6.6%	1.9%	2.3291	0.020 *
Professional literature	53	12	6.6%	7.5%	−0.4181	0.674
Friends	43	5	5.3%	3.1%	1.1806	0.238
Mass media (radio, TV)	29	5	3.6%	3.1%	0.3019	0.764
Cookbooks	9	15	1.1%	9.3%	−6.1202	<0.00001 ***
Newspapers and magazines (in paper)	4	1	0.5%	0.6%	−0.205	0.841
Other	17	6	2.1%	3.7%	−1.2379	0.215
**One of the three chief information sources**						
Experts, e.g., dietitians, physicians	385	110	47.6%	68.3%	−4.8061	<0.00001 ***
Websites managed by institutions	368	59	45.5%	36.6%	2.0641	0.039 *
Family	351	75	43.4%	46.6%	−0.7465	0.453
Social media, e.g., Facebook	313	24	38.7%	14.9%	5.7879	<0.00001 ***
Friends	232	40	28.7%	24.8%	0.9887	0.322
Bloggers	205	14	25.3%	8.7%	4.6131	<0.00001 ***
Professional literature	185	47	22.9%	29.2%	−1.7181	0.085
Mass media (radio, TV)	118	14	14.6%	8.7%	1.9907	0.047 *
Cookbooks	107	61	13.2%	37.9%	−7.552	<0.00001 ***
Newspapers and magazines (in paper)	51	11	6.3%	6.8%	−0.2502	0.80258
Other	91	24	11.2%	14.9%	−1.3114	0.1902

Note: Two-tailed hypothesis was tested. Statistically significant differences between groups were highlighted in bold (* *p* < 0.05, *** *p* < 0.001). PL group: n = 812, UK group: n = 161.

**Table 4:** The differences in significance of selected attributes of purchased food products between respondents from PL and the UK.

Product Attribute	Mean		*p*
	PL	UK	
The UK/PL provenance	3.44	3.76	0.000 ***
Food is produced in a traditional manner	3.30	3.25	0.387
The food is organically certified	3.14	3.22	0.380
Food products are produced in the region I live in	2.99	3.38	0.000 ***
The food is a ‘low food miles’ product	2.89	3.63	0.000 ***

Note: The average calculated on a rating scale from 1 (completely insignificant) to 5 (very significant). The Mann–Whitney U test was used to calculate the probability value (*p*). Statistically significant differences are highlighted in bold (*** *p* < 0.001).

**Table 5:** The differences in motivations for purchasing organic products between respondents from PL and the UK.

Motivations	Mean		*p*
	PL	UK	
Taking care of your health generally and/or health of your close family members	4.09	4.12	0.595
Wishing to eat higher-quality food products	4.06	4.16	0.127
Taking care of the environment	3.76	4.32	0.000 ***
Willingness to try something new	3.63	3.24	0.001 ***
You feel organic food is fresher than other foods	3.55	3.40	0.183
Wishing to live a specific lifestyle	3.52	3.19	0.007 **
A lack of confidence in conventional food	3.41	3.22	0.163
Your health problems and/or health problems of your close family members	3.07	3.03	0.804
The influence of what you have seen on social media	2.83	2.27	0.000 ***
The influence of people you live with	2.71	2.67	0.794
Organic food consumption has become fashionable	2.4	2.39	0.980

Note: The average calculated on a rating scale from 1 (strongly disagree) to 5 (strongly agree). The Mann–Whitney U test was used to calculate the probability value (*p*). Statistically significant differences were highlighted in bold (** *p* < 0.01, *** *p* < 0.001).

**Table 6:** The differences in frequency of purchasing certain organic product categories between respondents from PL and the UK.

Product Category	Mean		*p*
	PL	UK	
Eggs	3.65	3.91	0.223
Fresh fruits and vegetables	3.62	3.89	0.017 *
Olive oil	2.95	3.16	0.241
Meat and meat preparations	2.92	3.46	0.002 **
Dairy products	2.85	3.61	0.000 ***
Groats (e.g., buckwheat)	2.78	2.38	0.008 **
Coffee and tea	2.74	3.26	0.002 **
Pasta	2.72	2.91	0.307
Bread	2.69	2.85	0.246
Rice	2.49	2.76	0.079
Butter and margarine	2.46	3.22	0.000 ***
Frozen fruits and vegetables	2.34	2.41	0.713
Chocolate	2.11	2.84	0.000 ***
Cookies and pastries	2.05	2.23	0.064
Wine	2.01	2.47	0.000 ***
Crisps and salty biscuits	1.88	2.09	0.014 *
Sweets and candy	1.88	1.91	0.313
Soft drinks	1.84	1.85	0.289
Beer	1.79	1.83	0.082

Note: The average calculated on a rating scale from 1 (never) to 5 (always). The Mann–Whitney U test was used to calculate the probability value (*p*). Statistically significant differences were highlighted in bold (* *p* < 0.05, ** *p* < 0.01, *** *p* < 0.001).

## Data Availability

The data that support the findings of this study are available on request from M.R.

## Supplementary Materials

The following are available online at https://www.mdpi.com/article/10.3390/ijerph19031137/s1.
