# Supplementary material for: Comparison of the Purchasing Behaviour of Polish and United Kingdom Consumers in the Organic Food Market during the COVID-19 Pandemic
Source: Int J Environ Res Public Health. 2022 Jan 20;19(3):1137. doi: 10.3390/ijerph19031137 (PMC8834575; doi:10.3390/ijerph19031137)
Supplement: Supplementary file 1 [file ijerph-19-01137-s001.zip › ijerph-1556651-supplementary.pdf]

I PURCHASES OF FOOD (GENERAL)

1. How often do you do grocery shopping?

a. Every day
b. Several times a week
c. Once a week
d. Less than once a week
e. I don't do grocery shopping → thank you for participating in the survey

2. Where do you generally buy products belonging to the following food categories? Please tick all that apply. Please tick answers on each line.

	Hypermarket	Supermarket	Discount store, e.g. Lidl	Small format grocery store	Convenience store	Organic specialist store	Street or Farmers Market	Farm/farm shop (direct purchases from a farmer)	On-line shop e.g. internet sales or box scheme)	Other store	I make or grow this food myself	I do not buy these products
Fresh fruit and vegetables												
Meat and meat preparation												
Fish and fish preparation												
Eggs												
Honey												
Dairy												
Bread												

3. **When choosing food products, how important is the following attribute for you?**

	Totally unimportant	Not important	Neutral	Important	Very important
~~Polish/~~ The UK provenance					
Food products produced in the region I live					
The product labelling has a farm assurance or supply chain assurance label e.g. Red Tractor					
The food is organically certified					
Food is produced in a traditional manner					
The food is a 'low food miles' product					

II HEALTH

4. **What is healthy eating for you? Please address the following statements.**

	Strongly disagree	Disagree	Neutral	Agree	Strongly agree
Taking care of the quality of my food					
Taking care of the safety of my food					
Taking care of the provenance of my food					
Taking care of the freshness of my food					
Making sure that I eat foods that are good for me and avoiding products that are bad for me.					
Eating at appropriate meal times					
Taking care of the appropriate caloric balance of my food intake					
Taking care of the appropriate nutrient balance of my food intake					
Taking care to reduce the environmental impact of my food choices					
Ensuring that my diet is adapted to my lifestyle (physical activity, occupation etc.)					

5. **Do you think you eat healthily?**

a. No
b. I do not know
c. Yes

6. **Which are your main sources of information about food, diet and nutrition? Please choose the three most important sources of information and order them by importance.**

a. Family
b. Friends
c. Experts, e.g. dietitian, physician
d. Mass Media (TV, radio)
e. Institutional websites
f. Websites
g. Social media, e.g. Facebook
h. Bloggers
i. Specialized books
j. Recipe books
k. Periodicals and newspapers (paper version)
l. Other

III THESE QUESTIONS ARE ABOUT ORGANIC FOOD SPECIFICALLY

***7.* Do you buy organic certified food?**

Organic food is the term for food produced using organic farming methods. Such agriculture does not use, for example, artificial fertilizers or chemical plant protection products. Companies whose products can be called "eco" or "bio" must meet certain requirements, the fulfilment of which is confirmed by obtaining an appropriate certificate.

a. **No** → go to questions for this group of people
b. **Yes** → go to questions for this group of people

[the next questions are only for those who buy organic food]

8. What motivates you to buy organic food?

	Strongly disagree	Disagree	Neutral	Agree	Strongly agree
Taking care for your health generally and/or health of your close family members					
Your health problems and/or health problems of your close family members					
You feel organic food is fresher than other foods					
A lack of confidence in conventional food					
Taking care of the environment					
Organic food consumption has become fashionable					
Willingness to eat higher-quality food products					
Willingness to try something new					

Wishing to live a specific lifestyle					
The impact of people you live with					
The impact of what you have seen on social media					

9. How do you feel about/what is your attitude towards organic certified products? Please choose the most appropriate answer.

- I am sceptical in relation to organic food. I do not trust it
- I am cautious with regard to organic food. I buy certain organic products but not others.
- I am interested in buying organic food. I am buying more and more organic products.
- I am a keen advocate of organic food/ I am a staunch supporter of organic food

10. Which products do you buy as organic food and how often?

	Always	Often	Sometimes	Rarely	Never	I do not buy these foods
Dairy products						
Fresh fruit and vegetables						
Bread						
Eggs						
Groats (e.g. Buckwheat)						
Olive oil						
Frozen fruit and vegetables						
Chocolate						
Cookies and pastries						
Wine						
Beer						
Crisps and salty biscuits						
Sweets and candy						
Soft drinks						
Butter and margarine						
Meat and meat preparations						
Rice						
Pasta						
Coffee or tea						

11. Which foods are alternatives to organic certified food products that you buy or get? Please tick all that apply. The purchase may result from the non-availability of the product and / or the belief that there are products as good as organic food products or even better.

- Conventional food

Conventional food is generally available in stores and produced in a manner ensuring the highest possible production efficiency and profitability with the use of legally permitted fertilizers, pesticides, food additives, etc.

- Food products originating from your country
- Traditional and regional food
- Local food
- Products labelled as non-GMO

- Products with sustainability cues on packaging (e.g. Fairtrade, Rainforest Alliance)
- Vegetarian/ vegan food
- Food products obtained directly from a farmer (including family and friends)
- Food produced on your own land/garden
- None of the aforementioned food products

[the next questions are only for those who do not buy organic food]

8. Why don't you buy certified organic food?

	Strongly disagree	Disagree	Neutral	Agree	Strongly agree
The price of organic food is too high					
Low availability of organic products					
Insufficient labelling					
Inadequate promotion					
A lack of confidence in the certification					
No organic products available from your favourite food companies					
A lack of interest in organic food					
Self-production of food					

9. **What do you think about certified organic food? [open question]……………………………………… ……..**

10. **Which foods are alternatives to organic certified food products that you buy or get? Please tick all that apply.** The purchase may result from the non-availability of the product and / or the belief that there are products as good as organic certified food products or even better.
    - Conventional food

Conventional food is generally available in stores and produced in a manner ensuring the highest possible production efficiency and profitability with the use of legally permitted fertilizers, pesticides, food additives, etc.

- Food products originating from your country
- Traditional and regional food
- Local food
- Products labelled as non-GMO
- Products with sustainability cues on packaging (e.g. Fairtrade, Rainforest Alliance)
- Vegetarian/ vegan food
- Food products obtained directly from a farmer (including family and friends)
- Food produced on your own land/garden
- None of the aforementioned food products

11. Which products would you like to buy as organic?

	Definitely not	Probably not	It's hard to say	Probably yes	Definitely yes	I don't buy these products
Dairy products						
Fresh fruit and vegetables						
Bread						
Eggs						
Groats (e.g. Buckwheat)						
Olive oil						
Frozen fruit and vegetables						
Chocolate						
Cookies and pastries						
Wine						
Beer						
Crisps and salty biscuits						
Sweets and candy						
Soft drinks						
Butter and margarine						
Meat and meat preparations						
Rice						
Pasta						
Coffee or tea						

[the next questions are for all the respondents]

12. **What types of products** would you like to/ are you going to **buy more in the future?**

	I am not going to buy more	I would like to buy more	I am going to buy more
Certified organic food			
Local food			
Food products originated in your country			
Traditional and regional food			
Food products obtained directly from a farmer (including family and friends)			
Vegetarian/ vegan food			

Food products with sustainability cues on packaging (e.g. Fairtrade, Rainforest Alliance)			
Food produced on your own			

IV PERCEPTION OF DIFFERENT KINDS OF FOOD

13. In your opinion, conventional food is … Please address each pair of terms.

Unaffordable	1	2	3	4	5	Affordable
Unhealthy						Healthy
Untrustworthy						Trustworthy
Not good quality						Good quality
Not strictly controlled						Strictly controlled
Inauthentic						Authentic
Not available in stores						Available in stores
Not safe						Safe

14. In your opinion, certified organic food is … Please address each pair of terms.

Unaffordable	1	2	3	4	5	Affordable
Unhealthy						Healthy
Untrustworthy						Trustworthy
Not good quality						Good quality
Not strictly controlled						Strictly controlled
Inauthentic						Authentic
Not available in stores						Available in stores
Not safe						Safe

15. In your opinion, traditional and regional food is … Please address each pair of terms.

Unaffordable	1	2	3	4	5	Affordable
Unhealthy						Healthy
Untrustworthy						Trustworthy
Not good quality						Good quality
Not strictly controlled						Strictly controlled
Inauthentic						Authentic
Not available in stores						Available in stores
Not safe						Safe

16. In your opinion, local food is… Please address each pair of terms.

Unaffordable	1	2	3	4	5	Affordable
Unhealthy						Healthy
Untrustworthy						Trustworthy

Not good quality						Good quality
Not strictly controlled						Strictly controlled
Inauthentic						Authentic
Not available in stores						Available in stores
Not safe						Safe

17. In your opinion, food products originated in your country are … Please address each pair of terms.

Unaffordable	1	2	3	4	5	Affordable
Unhealthy						Healthy
Untrustworthy						Trustworthy
Not good quality						Good quality
Not strictly controlled						Strictly controlled
Inauthentic						Authentic
Not available in stores						Available in stores
Not safe						Safe

18. In your opinion, food products obtained directly from a farmer (including family and friends) are … Please address each pair of terms.

Unaffordable	1	2	3	4	5	Affordable
Unhealthy						Healthy
Untrustworthy						Trustworthy
Not good quality						Good quality
Not strictly controlled						Strictly controlled
Inauthentic						Authentic
Not available in stores						Available in stores
Not safe						Safe

19. In your opinion, vegetarian/ vegan food is … Please address each pair of terms.

Unaffordable	1	2	3	4	5	Affordable
Unhealthy						Healthy
Untrustworthy						Trustworthy
Not good quality						Good quality
Not strictly controlled						Strictly controlled
Inauthentic						Authentic
Not available in stores						Available in stores
Not safe						Safe

20. **In your opinion, food products with sustainable cues on packaging (e.g. Fairtrade, Rainforest Alliance) are … Please address each pair of terms.**

Unaffordable	1	2	3	4	5	Affordable
Unhealthy						Healthy
Untrustworthy						Trustworthy
Not good quality						Good quality
Not strictly controlled						Strictly controlled
Inauthentic						Authentic
Not available in stores						Available in stores
Not safe						Safe

21. **In your opinion, food produced on your own is … Please address each pair of terms.**

Unaffordable	1	2	3	4	5	Affordable
Unhealthy						Healthy
Untrustworthy						Trustworthy
Not good quality						Good quality
Not strictly controlled						Strictly controlled
Inauthentic						Authentic
Not available in stores						Available in stores
Not safe						Safe

V GREEN CONSUMPTION
[Questions concerning environmental attitude and behaviour]

22. **Please respond to the following statements**

    1 – strongly disagree
    2- disagree
    3 – neutral
    4 – agree
    5 – strongly agree

	1	2	3	4	5
**General Prosocial Attitudes**<br>1. It is important that others are happy<br>2. It is important to help someone who needs it<br>3. I want to help others<br>4. The well-being of others is important<br>5. The needs of others are important<br>6. It is important that all people are happy<br>**Green consumption values**<br>1. It is important to me that the products I use do not harm the environment<br>2. I consider the potential environmental impact of my actions when making many of my decisions<br>3. My purchase habits are affected by my concern for our environment<br>4. I am concerned about wasting the resources of our planet					

5. I would describe myself as environmentally responsible					
6. I am willing to be inconvenienced in order to take actions that are more environmentally friendly					
**Receptivity to green communication**					
1. I support brands that support the environment.					
2. I tend to pay attention to advertising messages that talk about the environment.					
3. The use of green messages in ads affects my attitude toward the ads.					
4. I respond favourably to brands that use green messages in their advertising.					
5. I am the kind of consumer who responds favourably when brands use green messages in their ads. I think that green advertising is valuable.					
6. Green advertising is a necessary form of advertising.					
7. I am the kind of consumer who is willing to purchase products marketed as being green.					
8. I tend to pay attention to green advertising messages.					
**Buying Behaviour**					
1. I try to buy energy efficient products and appliances					
2. I avoid buying products that have excessive packaging					
3. When there is a choice, I choose the product that causes the least pollution					
4. I have switched products/brands for ecological reasons					
5. I make every effort to buy paper products made from recycled paper					
6. I use environmentally friendly soaps and detergents					
7. I have convinced members of my family or friends not to buy some products which are harmful to the environment					
8. Whenever possible, I buy products packaged in reusable containers					
9. I try to buy products that can be recycled					
10. I buy high efficiency light bulbs to save energy					

RESPONDENT'S PARTICULARS

23. Gender
- a. Female
- b. Man

24. Age (years)
- **a. below 18**
- **b. 18-23 (Generation Z)**
- c. 24-39 (Millennials)
- d. 40-55 (Generation X)
- e. 55-73 (Boomers)
- f. 74-92 (Silent)
- g. **over 92**

25. Place of residence
- a. Cirencester
- b. Other

26. Are you a student?
- a. Yes
- b. No

27. Name of your university/ college
....

28. Your field of study: ……………………………………..

29. Perceived financial situation:

a. very good (I can afford to buy what I want and save/ invest some money)
b. good (I can afford to buy what I want)
c. average (I can afford to buy most of the things I would like to have)
d. bad (I am only able to meet my basic needs)
e. very bad (I am not able to meet my basic needs)

30. Nationality

a. UK
b. Other, ……………….

31. Do you have a food allergy?

a. No
b. Yes

Thank you!
